# The mediating role of psychological capital in the relationship between EFL learners’ L2 grit and L2 WTC

**DOI:** 10.3389/fpsyg.2025.1621340

**Published:** 2025-07-10

**Authors:** Xiaoming Chen, Areej Radhi Alruwaili, Mostafa Azari Noughabi, Ameneh Ghasemi, Changhui Zhen

**Affiliations:** ^1^School of Public and Basic Courses, Bengbu Medical University, Bengbu, China; ^2^Department of English Language, College of Arts, Jouf University, Sakaka, Saudi Arabia; ^3^Department of English Language and Literature, Hakim Sabzevari University, Sabzevar, Iran; ^4^Department of English Language and Literature, Al-Zahra University, Tehran, Iran

**Keywords:** L2 grit, psychological capital, L2 WTC, structural equation modeling, EFL learners

## Abstract

**Introduction:**

The key role of L2 grit has been acknowledged in English as a foreign language (EFL) learners’ linguistic and psychological development. However, there is still debate regarding the role of L2 grit in enhancing EFL learners’ willingness to communicate (WTC) in L2. Furthermore, due to the novelty of the concept of psychological capital (PsyCap) in the field of foreign language education, little is known about its mediating role in the relationship between L2 grit and WTC.

**Methods:**

The current study used valid, online questionnaires and employed structural equation modelling (SEM) to test whether Chinese EFL learners’ (*N* = 664) PsyCap can mediate the relationship between their domain-specific grit and WTC.

**Results:**

The results indicated that Chinese EFL learners’ PsyCap partially mediated the relationship between L2 grit and WTC. Therefore, L2 grit impacted EFL learners’ WTC in L2 both directly and indirectly through their positive psychological resources such as optimism, resilience, efficacy, and hope.

**Discussion:**

The findings highlight the criticality of positive psychological states in language classroom settings where EFL learners can enhance their tendency to communicate in L2 with higher levels of perseverance of effort and consistency of interest.

## Introduction

In recent years, success in a foreign or second (L2) language program has been increasingly measured through learners’ willingness to communicate (WTC) in L2 (Henry and MacIntyre, 2023). WTC is “an individual’s volitional inclination towards actively engaging in the act of communication in a specific situation, which can vary according to interlocutor, topic, and conversational context, among other potential situational variables” (Kang, 2005, p. 291). L2 WTC, the final psychological stage before actual L2 speaking (MacIntyre et al., 1998), plays an important role in learners’ L2 learning process (Henry and MacIntyre, 2023). In other words, the more willing the language learners are to communicate in L2, the more L2 achievements they have (Henry and MacIntyre, 2023). Therefore, to achieve success, language programs are expected to focus their attention on learners’ WTC in L2.

Since the advancement of positive psychology has directed more attention than ever to the influence of positive variables in the SLA research (e.g., Azari Noughabi and Ghasemi, 2024; Dewaele and Dewaele, 2018; Ghasemi and Azari Noughabi, 2024; MacIntyre et al., 2019), L2 WTC has been recently examined in terms of its relationship with positive variables (Ebn-Abbasi and Nushi, 2022; MacIntyre et al., 2019) which play a vital role in L2 communication (Wang, 2017). Before the positive psychology movement, the SLA literature was mostly overshadowed by negative affective variables such as anxiety (Ebn-Abbasi and Nushi, 2022). However, according to Arnold and Brown (1999), the question of negative affective variables has received much more attention and we should delve into the question of positive affective variables as well.

L2 grit is a positive affective factor for academic achievement (Botes et al., 2024) which is associated with L2 learners’ WTC (Ebn-Abbasi et al., 2024); in fact, gritty learners are more likely to display WTC in L2 due to their incessant inspiring efforts (Azari Noughabi and Ghasemi, 2024). In other words, learners with higher levels of L2 grit put more efforts in their language learning process, remain resilient despite challenges in communication in L2, and sustain their motivation to master their linguistic skills (Cheng, 2021; Derakhshan and Fathi, 2024; Lan et al., 2021). Thus, L2 grit can significantly predict learners’ L2 WTC (Lee and Drajati, 2019). Another positive variable linked to L2 WTC is psychological capital (PsyCap), one’s positive psychological state characterized by four resources, namely hope, efficacy, resilience, and optimism (Wu and Kang, 2025). PsyCap predicts vital indicators of learning-related outcomes, contributes to learners’ optimal functioning (Wu and Kang, 2025), and positively affects their academic performance (Carmona-Halty et al., 2021; Datu et al., 2018). Lin (2020) regards PsyCap as a key predictor of academic success, depositing from Luthans et al.’s (2007, 2010) statement about the influence of individuals’ positive psychological capacities on their performance. In addition, PsyCap drives gritty learners toward more successful academic performance through mediating the relationship between learners’ grit and their performance in academia (Luthans et al., 2019). According to Gordani and Sadeghzadeh (2023), “addressing psychological capital among students and examining its correlations is important” as “it seems that the existence of psychological capital among people, especially students, equips them with beliefs, faiths and attitudes which in turn can have positive consequences for their educational performance” (p. 1789), in this case L2 WTC.

Despite the key role of learners’ positive psychological capacities in their academic careers, scant research attention has been paid to the concept of PsyCap within the domain of L2 education (Hsu, 2024; Wu and Kang, 2025). Additionally, there is a scarce of research on the contribution of PsyCap in EFL learning contexts (Khajavy et al., 2019; Lin, 2020) where the domain-specificity of the PsyCap scale and the sources of EFL learners’ PsyCap have remained an untouched research territory until recently (Wu and Kang, 2025). Besides, given that most of the previous research explored this construct in western contexts, there is a need to explore PsyCap in collectivist societies such as Iran (Datu and Valdez, 2016, as cited in Khajavy et al., 2019). Furthermore, despite the crucial role of learners’ L2 WTC, L2 Grit, and PsyCap in their L2 learning; the relationship between these variables has not been explored, particularly within the field of L2 education. Part of this issue is because of the lack of a scale for measuring EFL learners’ PsyCap (Hsu, 2024). Finally, as Ebn-Abbasi and Nushi (2022) state “grit is a positive internal variable that has not been studied sufficiently in relation to SLA and, of course, L2 WTC, as much as other internal variables such as motivation and language aptitude” (p. 2).

Due to the critical role of WTC in facilitating learners’ L2 use (Kang, 2005), finding its correlates is significant. To this end, drawing on the principles of positive psychology and Broaden-and-Build Theory, and employing a domain-specific EFL PsyCap scale validated by Wu and Kang (2025), the present study seeks to model the relationships between English as foreign language (EFL) learners’ L2 grit and WTC in L2 with PsyCap as a mediator. Hence, this study aims to answer to what extent, EFL learners’ PsyCap can mediate the relationship between L2 grit and L2 WTC. In fact, our approach in hypothesizing the following model is in line with the objectives of L2 WTC theory (MacIntyre et al., 1998) and positive psychology (Seligman and Csikszentmihalyi, 2000) to depict the possible association between L2 WTC and positive affective attributes (L2 grit and PsyCap in this case). Wang and Wei (2024) recommend that researchers should carefully examine the relationship between independent variable and dependent variable before “focusing on generating additional explanations with mediators” (p. 549). Therefore, the current research aims to examine the mediating role of PsyCap in the relationship between EFL learners’ L2 grit and WTC which has been documented in earlier studies (Azari Noughabi and Ghasemi, 2024; Botes et al., 2024; Bensalem et al., 2023; Lan et al., 2021).

## Literature review

### Willingness to communicate (WTC)

**Figure 1:**
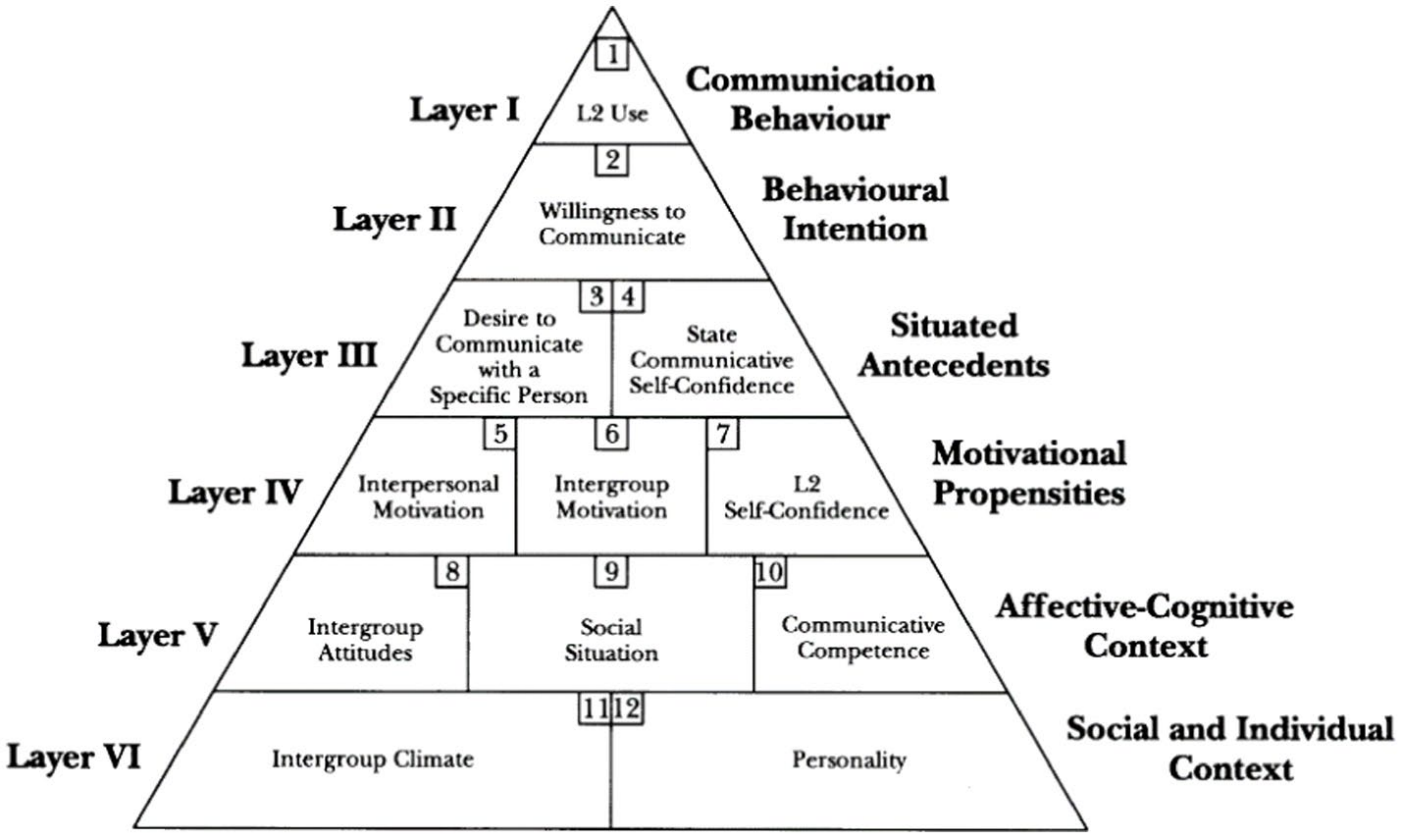
MacIntyre et al.’s (1998) heuristic model of L2 WTC.

The concept of WTC was initially introduced by McCroskey and Baer (1985) about one’s performance in first language (L1). It was described as a “personality-based, trait-like predisposition which is relatively consistent across a variety of communication contexts and types of receivers” (McCroskey and Baer, 1985, p. 6). Several years later, MacIntyre et al. (1998) claimed that L1 WTC cannot completely picture one’s WTC in L2. They defined L2 WTC as “a readiness to enter into discourse at a particular time with a specific person or persons, using an L2” (MacIntyre et al., 1998, p. 547). In a heuristic model of L2 WTC, MacIntyre et al. (1998) depicted the variables leading a person into sharing their opinion, knowledge, and thoughts with other individuals in an L2 (Figure 1). With regard to MacIntyre et al.’s (1998) Heuristic Model of L2 WTC, Figure 1 shows that WTC stands at Layer II, L2 grit belongs to Layer VI, and PsyCap can be related to Layers V and VI. MacIntyre et al.’s (1998) Heuristic Model of L2 WTC implies that language learners with similar language proficiency levels may have various levels of WTC due to individual differences, variety in their personality-based features, and a series of psychological or social factors.

This model comprises six layers where the top three (i.e., communication behavior, behavioral intention, and situated antecedents) expand the dynamic and situated perspectives of L2 WTC, and the bottom three (i.e., motivational propensities, affective-cognitive context, and social-individual context) provide the original trait-like views. In the present study, L2 WTC refers to how willing EFL learners are to communicate and interact in English at a particular time. Henry and MacIntyre (2023) strongly advocate that the research on WTC in English should concentrate on the role of different variables contributing to an increase or decrease in one’s L2 WTC.

Several studies have already reported significant relationships between L2 WTC and language learners’ affective variables such as anxiety and motivation (Lee and Hsieh, 2019; Lee and Lee, 2020), their emotional intelligence (Gao et al., 2025), their psychological resources such as hope and resilience (Khajavy et al., 2019), and personality related factors such as L2 grit (Derakhshan and Fathi, 2024). Lee and Lee (2020) conducted a quantitative study to examine how L2 WTC is influenced by affective factors (risk-taking, motivation, self-confidence, and speaking anxiety) and virtual intercultural experiences. The hierarchical regression analyses on the data from 176 Korean graduate and undergraduate EFL students revealed that learners with higher levels of L2 grit and motivation and lower levels of anxiety displayed a higher level of L2 WTC. In another recent study in the Iranian context, Ebn-Abbasi et al. (2024) found that EFL learners’ ideal L2 self and growth language mindset fully mediated the influence of L2 grit on their WTC.

### L2 grit

Grit is a personality characteristic which refers to “perseverance and passion for long-term goals” (Duckworth et al., 2007, p. 1087). According to Teimouri et al. (2022), L2 grit is comprised of two dimensions: (a) the perseverance of effort (PE) which is one’s capability of persevering despite adversity, and (b) the consistency of interests (CI) which is an individual’s capability of keeping interests alive while encountering failures or challenges.

The theoretical frameworks underlying the concept of grit are positive psychology (Derakhshan et al., 2022) and Broaden-and-Build Theory (Fredrickson, 2001): the first theory emphasizes a shift from the preoccupied negative attributes of individuals toward an understanding of their positive characteristics (Azari Noughabi et al., 2024). The second denotes that positive emotions can expand one’s momentary thought-action repertoire, thereby broadening their psychological and cognitive resources. Research on grit has revealed that grittier individuals tend to study and work more persistently and longer, resulting in higher academic and professional performances (Duckworth et al., 2007); thus, grit is a significant predictor of one’s success in academic or non-academic areas (Teimouri et al., 2022).

A line of inquiry has examined the impact of grit on L2 WTC. Lee and Drajati (2019) investigated the relationship between 183 Indonesian EFL learners’ L2 grit along with several other affective variables (self-confidence, L2 speaking anxiety, and motivation), L2 WTC, and their informal digital learning of English. The findings indicated that L2 grit was among the significant predictors of L2 WTC. In a similar study, Lee and Hsieh (2019) found that the grittier learners displayed higher L2 WTC in inside-class, outside-class, and digital language learning contexts. Lee (2022) investigated whether EFL learners’ grit and classroom enjoyment are associated with their L2 WTC and found that the PE dimension of grit and classroom enjoyment predicted all cohorts’ WTC in L2, while the CI dimension of grit did not predict L2 WTC among all samples.

Cheng (2021) examined how L2 grit and future L2 self-guides might predict motivated outcomes measured by L2 WTC. Cheng (2021) found that the PE component of grit showed a superior predictive power over learners’ L2 WTC, compared to the CI component. Lee and Taylor (2024) conducted a mixed-method study to investigate if positive constructs (L2 grit, growth mindset, and classroom enjoyment) and Extramural English can predict 160 primary school students L2 WTC in the classroom and out of class settings in Hong Kong. Hierarchical regression analyses showed that enjoyment, grit, and Extramural English predicted learners’ L2 WTC in class, while grit, growth mindset, and Extramural English predicted L2 WTC out of classroom. Azari Noughabi and Ghasemi (2024) investigated the mediating role of L2 grit on the relationship between 313 Iranian EFL learners’ WTC and their informal digital learning of English and found that L2 grit fully mediated the relationship between the two variables. In a recent study on the mediating role of enjoyment and anxiety in the relationship between grit and WTC of 238 Chinese junior high school students, Li (2024) found that PE and CI, as two aspects of grit, significantly predicted learners’ WTC. In addition, the enjoyment mediated the relationships between PE and WTC and between CI and WTC. In a recent study, Li (2025) found that PE correlated with Chinese undergraduate English learners’ meaning-focused and form-focused L2 WTC and anxiety mediated the relationship between learners’ CI and form-focused L2 WTC. In another recent study, Bensalem et al. (2025) found that Saudi EFL learners’ grit and enjoyment had significant influence on their WTC in blended learning environments.

After all, in spite of a multitude of studies on EFL learners’ WTC and grit (e.g., Derakhshan and Fathi, 2024; Lan et al., 2021; Lee, 2022; Sun et al., 2024), the factors that mediate the relationship between EFL learners’ domain-specific grit and L2 WTC has yet to be explored in various EFL contexts (Ebn-Abbasi et al., 2024; Li, 2025). In particular, scant research attention has been paid to exploring the mediating role of psychological capital as a psychological factor which has been recently introduced to the field of applied linguistics (Derakhshan and Azari Noughabi, 2024).

### Psychological capital (PsyCap)

The positive psychology movement, a shift against the negative psychological approaches, has been promoted since the beginning of the present century. As a result of this movement, Luthans (2002) named a novel concept called positive organizational behavior which refers to practicing and investigating one’s positive psychological capacities that can be measured and enhanced to improve occupational performance. Later on, this concept gave birth to another novel construct called PsyCap (Luthans et al., 2007) which describes one’s state of positive psychology rising to battle challenges and keep moving forward (King et al., 2020; Luthans et al., 2019). Therefore, PsyCap is derived from positive organizational behavior, and it lies within the framework of the positive psychology theory.

According to Luthans et al. (2007), PsyCap is comprised of four sub-constructs of hope (i.e., a person’s abilities and perseverance to choose pathways that can successfully lead them into their objectives), self-efficacy (i.e., one’s confidence to confront challenges and take actions to overcome those challenges), resilience (i.e., one’s ability to bounce back from failures and cope with adversity), and optimism (i.e., a person’s positive attitude toward life as well as belief in existence of solutions to problems). Luthans et al. (2007) state that a combination of these four components forms a core construct of PsyCap that is more influential in effect and broader than any of the subscales alone. Nevertheless, the four components of PsyCap represent state-like constructs that may develop and change over time (Luthans et al., 2019). In the present study, PsyCap refers to EFL learners’ positive psychological resources including optimism, resilience, hope, and efficacy.

Initial research on PsyCap was conducted in organizational settings (Luthans et al., 2004, 2006), using Psychological Capital Scale (Luthans et al., 2007) designed for employees. King and Caleon (2021) adapted this scale to make it relevant to the educational field, thereby developing the 16-item School Psychological Capital Scale. Since then, research on this construct in the educational setting has continued to emerge (e.g., Kang et al., 2021; Kang and Wu, 2022). Recently, Wu and Kang (2025) have examined the validity and reliability of the PsyCap scale in the EFL context. They reported excellent psychometric properties for the 16-item EFL PsyCap scale and verified that “EFL PsyCap is a hierarchical construct underpinned by its four first-order components of optimism, hope, resilience, and self-efficacy” (Wu and Kang, 2025, p. 645).

**Figure 2:**
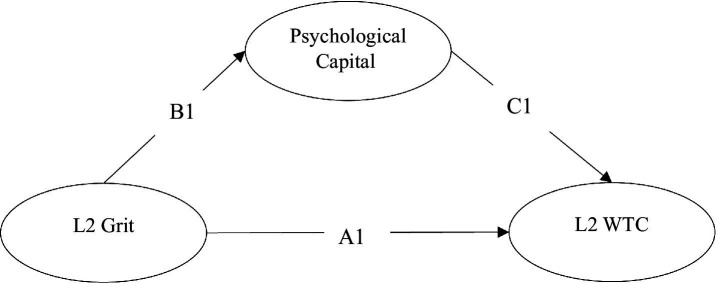
The hypothesized model of the present study.

A review the related literature suggests that a few studies have examined EFL learners’ PsyCap. Khajavy et al. (2019) conducted a study to investigate the role of 317 Iranian EFL learners’ PsyCap in their WTC in L2, L2 achievement, and L2 motivational self-system. Based on the results of structural equation modeling, PsyCap positively and significantly predicted the learners’ L2 WTC, L2 achievement, and L2 motivational self-system, thereby confirming the major impact of PsyCap in L2 education. Luthans et al. (2019) explored the mediational role of PsyCap in the relationship between students’ grit and their academic performance. The findings revealed that PsyCap was a significant mediator of the relationship between learners’ grit and academic performance. Nevertheless, as mentioned earlier, not until recently, have the prediction of PsyCap resources and the domain-specificity of its scale been investigated in an EFL context. In a recent attempt, Wu and Kang (2025) validated the 16-item EFL PsyCap scale, developed by King and Caleon (2021). Furthermore, their study indicated that EFL PsyCap resources positively predict EFL performance, behavioral engagement and academic enjoyment, while negatively predicting academic anxiety and boredom. All in all, a review of the related literature indicates that there is a dearth of research to explore the associations among EFL learners’ WTC, L2 grit, and psychological capital, using a domain-specific PsyCap scale. Therefore, this study attempts to shed light on this under-explored area of research. Figure 2 displays the hypothesized model of the current study.

## Methods

### Participants

A total of 664 Chinese EFL learners, the majority of whom were female (77.4%), took part in this research. The majority of the participants were English major students. They were from a wide age range (from 15 to 49 years old). In addition, most of the participants were undergraduate EFL learners. They were initially requested to report their demographic information and sign the informed consent form. Next, they were expected to respond to the items of three questionnaires that measured their L2 grit, L2 WTC, and PsyCap in L2 learning. 58.7% of the participants were English major students, followed by non-English major students (35.5%). 5.7% of the participants were not educating in universities. Table 1 demonstrates the demographic information of the participants.

**Table 1:** Demographic information of the participants.

Variable	Frequency (Percent)
Gender	Male = 150 (22.6%)
	Female = 514 (77.4%)
Age range	15–19 = 323 (48.6%)
	20–29 = 323 (48.6%)
	30–39 = 16 (2.4%)
	40–49 = 2 (0.3%)
Education level	Undergraduate = 583 (87.7%)
	Postgraduate = 68 (10.2%)
	Doctorate = 5 (0.8%)
	Others = 8 (1.2%)
Major	English major = 398 (58.7%)
	Non-English major = 236 (35.5%)
	Others = 38 (5.7%)

### Instruments

#### L2 grit scale

Teimouri et al.’s (2022) L2 Grit Scale was utilized to measure the EFL learners’ grit. This scale has a two-fold structure, including nine items: five items constitute the PE dimension of the grit scale (e.g., “I put much time and effort into improving my English language weaknesses.”) and four items constitute the CI dimension of this scale (e.g., “I have been obsessed with learning English in the past but later lost interest.”). Furthermore, the items of this questionnaire are rated on a 5-point Likert scale from 1 (*Not like me at all*) to 5 (*Very much like me*). Good construct validity and high internal consistency coefficients (0.66 for the CI sub-scale as well as 0.86 for the PE sub-scale) have been reported by Teimouri et al. (2022) for this instrument. In this study, the internal consistency coefficient for this instrument was reported to be 0.900.

#### Psychological capital scale

To measure the EFL learners’ PsyCap, the 16-item School Psychological Capital Scale was adapted. This scale has been constructed by King and Caleon (2021), and validated by Wu and Kang (2025) for the EFL context. Wu and Kang (2025) adapted all of the items of this scale so that they would be relevant to EFL learning. As an example, the original item in the optimism subscale, “I am optimistic about my future in school,” was changed into “I am optimistic about my future in English learning.” The following includes sample items for each of the other subscales: “I feel confident participating in English class discussions” (4-item self-efficacy subscale), “If I have problems in English learning, I could think of many ways to solve them” (4-item hope subscale), and “I am good at dealing with setbacks in English at school” (4-item resilience subscale). In addition, the items of this questionnaire are rated on a 7-point Likert scale from (1 = strongly disagree) to (7 = strongly agree). Besides, in the present study, the Cronbach’s *α* of this instrument was reported to be 0.930.

#### Willingness to communicate in English scale

To measure the participants’ L2 WTC, Peng and Woodrow’s (2010) WTC in English Scale was utilized. This scale consists of ten items (e.g., “I am willing to give a short speech in English to other people about my hometown with notes”) on a one-factor structure. Furthermore, a 5-point Likert scale ranging from 1 (*definitely not willing*) to 5 (*definitely willing*) is used to rate the items of this questionnaire. Peng and Woodrow (2010) have reported high validity and reliability for this scale (GFI = 0.97, CFI = 0.98, RMSEA = 0.07, α = 0.89). Moreover, Cronbach’s alpha reliability coefficient of this instrument was reported to be 0.920 in the present study.

### Data collection

The data for this study were collected from Chinese EFL learners. The participants were kindly invited to voluntarily take part in this research by completing an online survey formed and designed through *Wenjuanxing* (the equivalent of SurveyMonkey in China). The link of the survey, which contained one part for demographics and three scales for the three variables, was shared with the participants through WeChat and email. Snowball sampling was obtained to recruit the sample. Initially, the participants were expected to sign the informed consent form while they were assured that both their responses and identities would be kept anonymous and confidential. Furthermore, the items of the three scales utilized in this study were written in the English language, and no randomization of the items was involved for each participant. The data for this study were collected from March 2024 to April 2024 while the participants could take as long as they wished to fill out the questionnaires.

### Data analysis

Initially, the data were analyzed for the sake of ensuring a lack of missing value as well as data normality. After that, the researchers used confirmatory factor analysis (CFA) to test how well the variables represented the number of constructs. In addition, partial least squares structural equation modeling was conducted via AMOS 24 to focus on predicting variables by identifying the relationships between observed variables and underlying constructs (Hair and Alamer, 2022). Next, to assess model fit (Bentler, 2007), several indices were used: Tucker Lewis index (TLI), the goodness of fit (GFI), the chi-square/df ratio (χ^2^/df), the comparative fit index (CFI), standardized root mean square error of approximation (SRMR), and root mean square error of approximation (RMSEA). To achieve a good model fit, the values of SRMR and RMSEA should be less than 0.08 and the values of TLI and CFI should be above 0.90 (Bentler, 2007). Furthermore, mediation parameters suggested by Zhao et al.’s (2010) were considered.

In the present study, a mediation model of the relationship between L2 grit and WTC was hypothesized with PsyCap as the mediator. The hypothesized model of the current research is depicted in Figure 2. The following research hypotheses were made:

1. EFL learners’ L2 grit can significantly influence their L2 WTC.
2. EFL learners’ L2 grit can significantly influence their PsyCap.
3. EFL learners’ PsyCap can significantly influence their L2 WTC.

Mediation analysis was conducted to test the direct (A1) and indirect (B1 + C1) effects of L2 grit on L2 WTC. A partial mediation occurs when both direct and indirect effects are significant. Full mediation is claimed when only indirect path is statistically significant. To run the mediation analysis in the present study, a maximum likelihood estimator with a 5,000-sample bootstrapping (95% confidence interval) was utilized.

## Results

### Descriptive statistics

Descriptive statistics, shown in Table 2, revealed that the skewness and kurtosis values were acceptable in range (±2), thereby confirming the normal distribution of the data (Kunnan, 1998).

**Table 2:** Descriptive statistics and correlation matrix.

	L2 grit	PsyCap	L2 WTC
Descriptives			
Minimum	1.78	2.63	1.80
Maximum	5.00	7.00	5.00
Mean	3.399	4.736	3.437
Standard deviation	0.605	0.810	0.643
Skewness	0.063	0.198	−0.131
Kurtosis	0.109	−0.018	−0.113
Correlations			
L2 grit	1	–	–
PsyCap	0.710^***^	1	–
L2 WTC	0.523^***^	0.558^***^	1

PsyCap = psychological capital. ^***^p < 0.001.

Table 2 also presents the correlation coefficients between EFL learners’ L2 grit, PsyCap, and WTC. The calculated estimates showed significant and linear relationships between the variables. Furthermore, given that the correlations were not too high (*r* > 0.80), there was no possibility of multicollinearity (Field, 2013). Additionally, the VIF values did not surpass 2.014.

#### Confirmatory factor analysis results

CFA was run to examine the facture structure of the observed variables. Table 3 indicates the results of CFA for each main variable.

**Table 3:** CFA results.

	ꭓ^2^/df	GFI	CFI	TLI	SRMR	RMSEA
L2 grit	3.017	0.979	0.985	0.971	0.061	0.062
PsyCap	3.099	0.939	0.960	0.947	0.062	0.068
L2 WTC	2.924	0.990	0.994	0.978	0.074	0.077

PsyCap, psychological capital.

As shown in Table 3, model-fit indices were acceptable in range (Bentler, 2007). In addition, the results indicated that estimated factor loadings were beyond cut-off point (>0.50, suggested by Kline, 2016). Additionally, the calculated Average Variance Extracted (AVE) values were beyond 0.70 suggesting convergent validity (Fornell and Larcker, 1981). Moreover, AVEs were lower than composite reliability (CR) measures, thereby confirming the discriminant validity of the variables (Kline, 2016).

#### Mediation model analysis results

**Figure 3:**
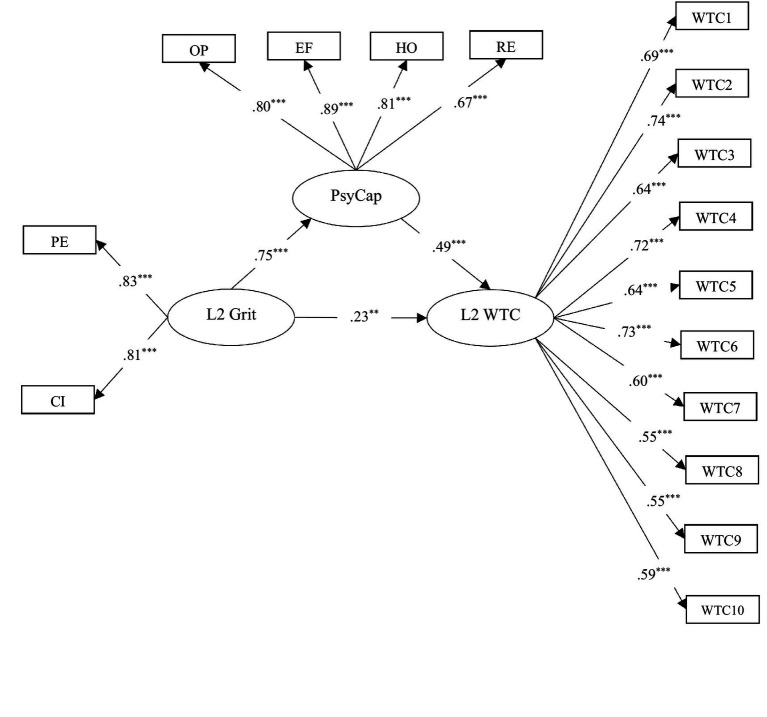
The mediation model of the relationships between L2 grit, psychological capital, and WTC. PE = perseverance of effort, CI = consistency of interest, OP = optimism, EF = efficacy, HO = hope, RE = resilience. ^***^p < 0.001, ^**^p < 0.01.

To test the mediating role of PsyCap in the relationship between EFL learners’ domain-specific grit and L2 WTC, the hypothesized mediation model was examined via SEM. The initial model fit indices were acceptable but the chi-square/df ratio; *χ*^2^/*df* = 3.420, GFI = 0.931, CFI = 0.945, TLI = 0.947, RMSEA = 0.075, *p* = 0.000. To achieve a better model-fit, the error terms of several items of WTC (Item 2 and Item 3; Item 8 and Item 9), whose item wordings were similar, were correlated (Brown, 2015). The results of SEM analysis showed a partial mediation model with an acceptable model fit (Kline, 2016); *χ*^2^ = 257.622, *df* = 91, *χ*^2^/*df* = 2.831, GFI = 0.953, CFI = 0.976, TLI = 0.968, SRMR = 0.057, RMSEA = 0.053, *p* = 0.000. The mediation model is shown in Figure 3.

Table 4 includes the results of direct and indirect effects.

**Table 4:** The results of mediation model.

	Estimate	*p*-value
Standardized direct effects		
L2 Grit → L2 WTC	0.228	0.000
L2 Grit → PsyCap	0.749	0.000
PsyCap → L2 WTC	0.492	0.000
Standardized indirect effects		
L2 Grit → PsyCap → L2 WTC	0.417	0.000

PsyCap = psychological capital.

As shown in Table 4, in addition to the significant effect of learners’ L2 grit on their WTC (*β* = 0.228), it was figured out that L2 grit had a significant influence on EFL learners’ PsyCap (*β* = 0.749). Given that PsyCap had a significant effect on EFL learners’ WTC (*β* = 0.492), it was identified as a partial mediator of the relationship between L2 grit and WTC among Chinese EFL learners (*β* = 0.417). Due to the significant effect of L2 grit on PsyCap, PsyCap on L2 WTC, and L2 grit on L2 WTC, a complementary mediation is announced (Zhao et al., 2010).

## Discussion

The current study aimed to investigate the role of PsyCap in mediating the relationship between EFL learners’ domain-specific grit and L2 WTC in the context of China. The results of SEM analysis revealed that EFL learners’ PsyCap was a partial mediator of the relationship between L2 grit and WTC. In addition, it was revealed that L2 grit could significantly affect EFL learners’ tendency to communicate in L2. In line with the underlying principles of positive psychology (MacIntyre et al., 2019) and Broaden-and-Build Theory (Fredrickson, 2001), the results of the present study highlighted the crucial role of positive psychological resources in facilitating the linguistic achievement of language learners. From a theoretical perspective, creating a positive loop for language learners who are striving with perseverance to enhance their communicative skills is beneficial (Derakhshan, 2022; Dewaele et al., 2023; Lee, 2022) given that positive psychological resources can offer learners a potential to navigate negative emotions, sustain a state of hopefulness, remain optimistic with a growth mindset, and tackle the challenges resiliently (Wu and Kang, 2025).

Regarding the first research hypotheses, the findings indicated that L2 grit significantly influenced EFL learners’ WTC. The significant role of L2 grit in EFL learners’ WTC is in support of earlier studies (Azari Noughabi and Ghasemi, 2024; Lee, 2022; Lee and Lee, 2020) suggesting that EFL learners with more perseverance and passion for long-term goals would tend to look up for various chances to communicate in L2 despite the challenges they may encounter in the odyssey of language learning (Yang et al., 2022; Botes et al., 2024). From a theoretical point of view, positive personality features can broaden one’s though-action repertoire which subsequently facilitates learning (Ebn-Abbasi et al., 2024; Li, 2025). This is in support of earlier studies (e.g., Botes et al., 2024) showing that when a language learner sustains his or her gritty behaviors, it is more likely that he or she enjoys a growth language mindset and accomplish language learning tasks. The findings were also in contrast with the study of Ebn-Abbasi et al (2024) which showed that L2 grit was indirectly linked to WTC through the mediating role of ideal L2 self and growth language mindset. This inconsistency reveals that there are still strong mediators in the relationship between L2 grit and WTC which need to be identified. This calls for further studies on the mediating mechanisms that guide EFL learners’ grit to blossom into successful communication in L2.

The second research hypothesis was confirmed suggesting that EFL learners’ L2 grit impacted their PsyCap. In fact, one of the novel findings was the significant role of L2 grit in enhancing EFL learners’ PsyCap. The relationship between L2 grit and PsyCap can be explained by the significant association of EFL learners’ domain-specific grit with self-efficacy (Azari Noughabi et al., 2024; Derakhshan and Fathi, 2023) which is regarded as one of the components of PsyCap (Wu and Kang, 2025). Although previous studies have mostly focused on PsyCap as a predictor of academic engagement in general education settings (Datu et al., 2018), the present study considered the mediating potential of PsyCap among EFL learners and revealed that L2 grit can act as a motivating drive which also sparkles the positive psychological resources (i.e., HERO standing for hope, efficacy, resilience, and optimism) that one has. It can be explained by the fact that gritty EFL learners are less likely to give up while facing hurdles in the process of language learning (Derakhshan and Fathi, 2024). Therefore, a gritty EFL learner who seeks to achieve his or her goals needs to address the difficulties embedded in the process of language learning by activating a positive mechanism consisting of hope, efficacy, resilience, and optimism. Accordingly, L2 grit would trigger students’ PsyCap, especially through the promotion of a HERO framework.

The third research hypothesis was also confirmed showing that EFL learners’ PsyCap significantly influenced their WTC. The significant influence of PsyCap on EFL learners’ WTC is in line with the study of Khajavy et al. (2019) which signified the crucial role of positive psychological factors in developing language learners’ WTC and motivational self-system. Therefore, not only do linguistic and cognitive variables affect L2 WTC, but also learners’ individual differences and psychological features can play an important role in the way learners become inclined toward communicating in L2 in their language classes (Henry and MacIntyre, 2023). In line with previous research which highlighted the significant relationship between WTC, positive affect (Alrabai, 2024; Lee and Taylor, 2024), and positive psychological properties such as hope, efficacy, resilience, and optimism (Khajavy et al., 2019); this study revealed that more positive psychological resources can increase tendency to communicative in L2. One point worthy to mention is that the current study considered PsyCap as a unified variable rather than a set of separated factors. Therefore, the EFL learners with higher levels of PsyCap and more positive psychological resources are likely to take various chances for success in communicating in L2.

### Implications

The findings of the present study implied the criticality of positive psychological resources in developing WTC. Accordingly, EFL learners need to become aware of the positive psychological resources they can use to direct their practices to learning English. EFL teachers are recommended to consider their students’ psychological potentials and have specific plans for improving these positive capacities. Positive psychological interventions can be one useful option for promoting EFL learners’ hope, efficacy, resilience, and optimism. In addition, it would be beneficial to introduce coping strategies for addressing challenges in the path of successful communication in L2. Another implication is to cultivate a growth mindset among EFL learners in classroom settings with the ultimate aim of promoting hope and optimism as two cornerstones of PsyCap. One way to do so can be encouraging EFL learners to share their positive experiences that had yielded positive outcomes. Reflecting on past experiences can also help learners believe that success is inherently interwoven with ups and downs. Incorporating reflective journaling into English classes is recommended as it can help EFL learners manage their anxiety and stress, particularly in speaking tasks.

The findings also imply that grit will result in higher L2 WTC if language learners’ psychological resources are summoned. EFL teachers can initially introduce easier communicative tasks and gradually increase the difficulty level of the tasks. EFL teachers can foster their students’ self-efficacy through achievable challenges by designing proper tasks beyond their current level of language proficiency and scaffolding them with personalized support. When most of the learners are able to complete L2 communicative tasks, they achieve a sense of accomplishment which in turn can promote their efficacy and hope. Therefore, they will see that their efforts are going to have a happing ending. Furthermore, EFL teachers can model the way a student can address communicative challenges by applying various learning strategies. Unfortunately, EFL learners cannot handle L2 tasks due to unfamiliarity with communicative skills and strategies. Additionally, EFL teachers can ask the learners and their peers to create a list of possible solutions for the challenges they face during communicating in L2. Finally, creating a loving classroom environment where teachers care about their learners, use a sense of humor, react to learners’ mistakes with kindness, and encourage team work activities can enrich learners’ positive psychological resources and increase their tendency to interact in L2.

## Conclusion

The purpose of the present study was to explore whether Chinese EFL learners’ PsyCap could mediate the relationship between their L2 grit and L2 WTC. The findings revealed that PsyCap was a partial mediator of this relationship. Accordingly, L2 grit was a strong predictor of WTC in L2 in the Chinese EFL context as it affects learners’ tendency to communicate in L2 both directly through passion and perseverance for achieving long-term goals and indirectly through enhancing their hope, resilience, efficacy, and optimism. This study suggests that EFL learners’ WTC would develop if EFL learners sustain their effort and interest during the learning process through their positive psychological resources which may act as an armoring mechanism against adversities and stressors. Finally, as they say “it takes two to tango,” positive personality features coupled with positive psychological resources are what EFL learners need to communicate in L2.

The current study had some limitations which should be acknowledged here. First, this study used self-report survey data to test the hypothesized relationships. Future studies can use data triangulation to provide a more vivid picture of the associations among the main variables of this study. Second, the role of demographic variables such as learners’ age, language learning experience, language proficiency level, and gender in the proposed model were not explored. Future research can open up a new line of inquiry that examines whether demographic factors make a difference in the way L2 grit affects L2 WTC. In addition, it is recommended to propose a more complex model by adding more relevant variables to the model. Third, the present study only focused on a sample of Chinese EFL learners. It is recommended to conduct cross-cultural studies to highlight the role of cultural factors that may affect the way learners understand their psychological resources. In addition, comparative studies are needed to check how contextual factors influence EFL learners’ psychological states as well as the way they put effort into their language learning. Finally, the present cross-sectional study did not embed a model in the longitudinal format to explain causality in a long period of time. Future studies can employ a growth curve modelling approach in order to observe the fluctuations across the variable relationships.

## Data Availability

The raw data supporting the conclusions of this article will be made available by the authors, without undue reservation.
